# The Effect of Exercise Training Modality on Serum Brain Derived Neurotrophic Factor Levels in Individuals with Type 2 Diabetes

**DOI:** 10.1371/journal.pone.0042785

**Published:** 2012-08-06

**Authors:** Damon L. Swift, Neil M. Johannsen, Valerie H. Myers, Conrad P. Earnest, Jasper A. J. Smits, Steven N. Blair, Timothy S. Church

**Affiliations:** 1 Department of Preventive Medicine, Pennington Biomedical Research Center, Baton Rouge, Louisiana, United States of America; 2 Behavioral Medicine Laboratory, Pennington Biomedical Research Center, Baton Rouge, Louisiana, United States of America; 3 Sport, Health and Exercise Science, Department for Health, University of Bath, Bath, United Kingdom; 4 Department of Psychology, Southern Methodist University, Dallas, Texas, United States of America; 5 Department of Exercise Science and Department of Epidemiology and Biostatistics, University of South Carolina, Columbia, South Carolina, United States of America; University of Granada, Spain

## Abstract

**Introduction:**

Brain derived neurotrophic factor (BDNF) has been implicated in memory, learning, and neurodegenerative diseases. However, the relationship of BDNF with cardiometabolic risk factors is unclear, and the effect of exercise training on BDNF has not been previously explored in individuals with type 2 diabetes.

**Methods:**

Men and women (N = 150) with type 2 diabetes were randomized to an aerobic exercise (aerobic), resistance exercise (resistance), or a combination of both (combination) for 9 months. Serum BDNF levels were evaluated at baseline and follow-up from archived blood samples.

**Results:**

Baseline serum BDNF was not associated with fitness, body composition, anthropometry, glucose control, or strength measures (*all*, p>0.05). Similarly, no significant change in serum BDNF levels was observed following exercise training in the aerobic (−1649.4 pg/ml, CI: −4768.9 to 1470.2), resistance (−2351.2 pg/ml, CI:−5290.7 to 588.3), or combination groups (−827.4 pg/ml, CI: −3533.3 to1878.5) compared to the control group (−2320.0 pg/ml, CI: −5750.8 to 1110.8). However, reductions in waist circumference were directly associated with changes in serum BDNF following training (r = 0.25, p = 0.005).

**Conclusions:**

Serum BDNF was not associated with fitness, body composition, anthropometry, glucose control, or strength measures at baseline. Likewise, serum BDNF measures were not altered by 9 months of aerobic, resistance, or combination training. However, reductions in waist circumference were associated with decreased serum BDNF levels. Future studies should investigate the relevance of BDNF with measures of cognitive function specifically in individuals with type-2 diabetes.

## Introduction

Brain derived neurotrophic factor (BDNF) is a protein encoded by the BDNF gene. It is a neutrophin (e.g., growth factor) involved in neuronal plasticity, differentiation, and survival in the central and peripheral nervous system [1], [2]. Low levels of BDNF have been associated with learning/cognitive dysfunction [2], depression [3], neurodegenerative conditions [4], and mortality [5]. BDNF has been studied in various populations; however, the clinical significance of BDNF levels remains unclear in individuals with type 2 diabetes. Previous studies have found that serum [6] and plasma [7] BDNF levels are lower in individuals with type 2 diabetes compared to non-diabetic individuals, posing the question of whether the higher rate of cognitive impairments in diabetes may be in part mediated by low BDNF levels [7]. Similar findings have been reported in non-diabetic individuals in which lower serum BDNF levels have been associated with insulin resistance and higher body fat [8].

Conversely, other studies have found the opposite relationship, noting elevated serum BDNF levels in individuals with type 2 diabetes [9]. In addition, higher serum and plasma BDNF levels have been associated with cardiometabolic risk factors [9]–[11], and specifically serum BDNF has been inversely correlated with total daily energy expenditure [12] and aerobic fitness [13], [14]. Fujinami et al. [6] found that serum BDNF was directly associated with fasting insulin and homeostatic model assessment of insulin resistance in women but not men in a Japanese sample (N = 112) of individuals with type 2 diabetes. It has been proposed that BDNF may be elevated in individuals with type 2 diabetes as a compensatory mechanism to provide additional neuroprotection due to the presence of chronic hyperglycemia and other cardiometabolic risk factors [9], [15].

Exercise training increases fitness [16], improves glycemic control [16], [17], and reduces body fat [16], [17] in individuals with type 2 diabetes. Specifically, the combination of aerobic and resistance training (combination training) has been shown to be the most effective in improving glycemic control compared to aerobic or resistance training alone [16]–[18]. Previous data on the effect of exercise training on BDNF levels is unclear with some evidence of higher plasma [19] and serum [20] BDNF levels following aerobic training while other studies have found no significant change in BDNF levels following aerobic [21]–[24] or resistance training [21]. However, studies in this area have important limitations/considerations such as small sample size [19], [22], no control group [19], were conducted in young healthy individuals [19], [21], [24], [25] or subject populations with specific neurological conditions [20], [26]. To our knowledge, the effects of exercise training or a comparison of different training modalities (aerobic, resistance, or a combination of both) on BDNF levels have not been explored in individuals with type 2 diabetes in a large randomized trial. The objectives of the present study were to (1) evaluate the association between cardiometabolic risk factors (fitness, anthropometry, body composition, and glucose control) and strength variables on baseline serum BDNF levels; and (2) to test the effects of aerobic, resistance, and combination exercise training on serum BDNF levels in individuals with type 2 diabetes.

## Methods

**Figure 1:**
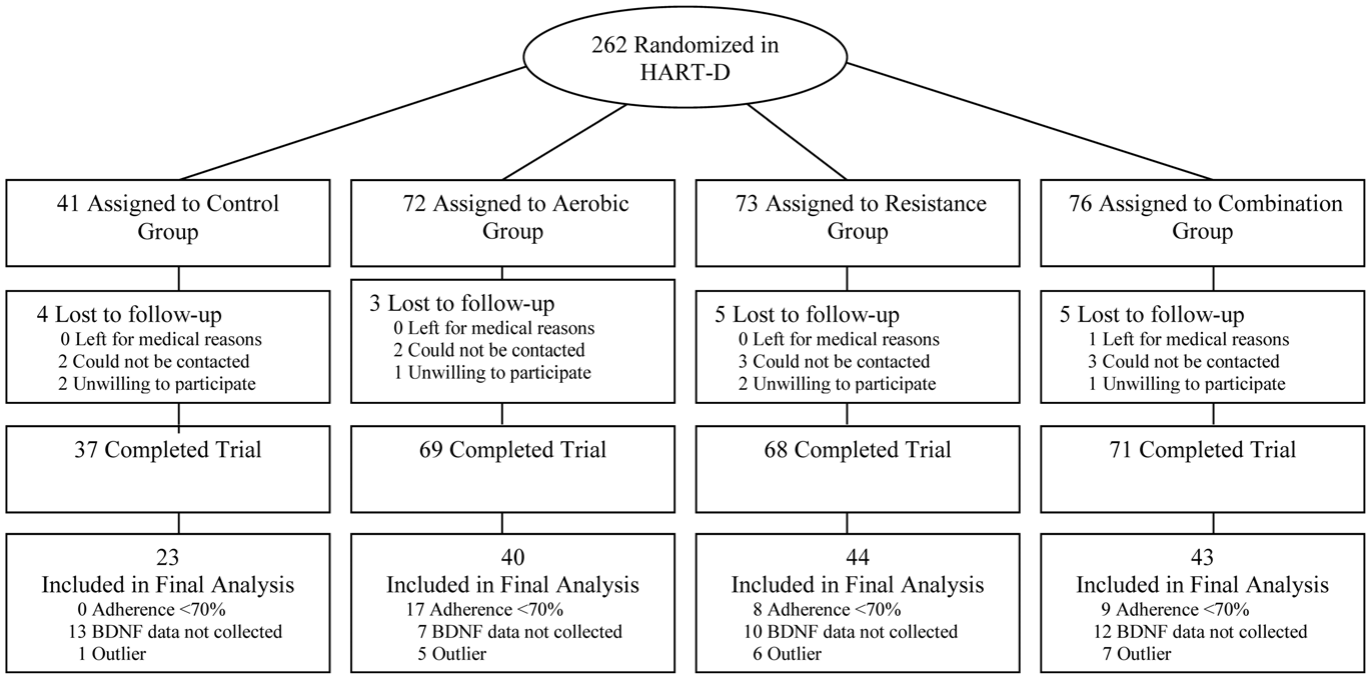
Consort diagram.

The full methodology of the exercise training program used in the Health Benefits of Aerobic and Resistance Training in Individuals with Type 2 diabetes (HART-D) has been reported in the main outcomes paper [16]. HART-D was a 9 month exercise study comparing the effects of aerobic training, resistance training, or a combination of both on HbA_1C_ in men and women with type 2 diabetes. The primary outcome measure of this ancillary study is the change in serum BDNF levels following exercise training. From the parent HART-D study of 262 sedentary (30–75 years) adults with type 2 diabetes presenting with an HbA_1C_ of 6.5% to 11.0%, serum BDNF values were examined in a subset of controls and adherent exercisers (n = 168) from which a total of 150 participants were included in the final analysis. A consort diagram is shown in Figure 1. Notable exclusion criteria for the HART-D trial included a body mass index (BMI)>48 kg/m^2^, blood pressure 160/100 mmHg or higher, fasting triglycerides 500 mg/dL or higher, use of an insulin pump, urine protein greater than 100 mg/dL, history of stroke, advanced neuropathy, retinopathy, or any serious medical condition that prevented adherence to the study protocol or was a contraindication for exercise training. The institutional review board at Pennington Biomedical Research Center approved the protocol annually and all participants provided written informed consent before initiating the any of the study protocols.

### Study Design

Participants were randomized to one of four groups: a non-exercise control group, aerobic training only (aerobic), resistance training only (resistance), or a combination of resistance and aerobic training (combination). The non-exercise control group was offered weekly stretching and relaxation classes and were asked to maintain their current activity during the 9-month study period. Accordingly, the compliance of the control group was assessed with step counters to confirm continued sedentary behavior. All exercise sessions were supervised by study staff in our exercise training laboratory.

The exercise intensity for aerobic exercise training was 50% to 80% of maximal oxygen consumption. We estimated that 150 minutes of physical activity per week (consistent with public health recommendations) is equivalent to approximately 10 to 12 kcals/kg of body weight per week (KKW) (5). The selected dose of exercise for the aerobic group was 12 KKW and 10 KKW for the combination exercise group. Participants were weighed weekly to calculate the energy expenditure target for each participant. American College of Sports Medicine equations were used to estimate caloric expenditure rate and time required for each session (1).

Participants in the resistance training group exercised 3 days per week with each session consisting of 2 sets of 4 upper body exercises (bench press, seated row, shoulder press and lat pull down), 3 sets of 3 leg exercises (leg press, extension, and flexion), and 2 sets of each abdominal crunches and back extensions. Each set of resistance exercise consisted of 10 to 12 repetitions. Individuals in the combination exercise group had two sessions per week with each session consisting of 1 set of each of the 9 resistance exercises. The weight lifted on each exercise was progressively increased once the participant was able to complete 12 repetitions for each set of exercises on 2 consecutive exercise sessions.

The control group was unblinded after a significant number of participants (17.1%) had an increase in HbA_1C_ level of 1.0% or higher. With the recommendation of the data safety monitoring board, randomization into the control group was discontinued (5). Thus, fewer participants completed the study in the control group compared to the other randomization groups.

### Exercise Testing

Exercise testing was performed on a treadmill (Trackmaster 425, Newton Kansas) with respiratory gasses analyzed using a True Max 2400 Metabolic Measurement Cart (Parvomedics, Salt Lake City Utah). Participants self-selected a brisk walking pace at a grade of 0% for 2 minutes, and then the grade was increased by 2% every 2 minutes until exhaustion. Fitness data are reported in absolute VO_2 peak_ (L/min), relative VO_2 peak_ (mL·kg^−1^·min^−1^), and treadmill test duration (min). All muscular strength assessments were performed using a Biodex System 3 dynamometer (Biodex Medical Systems, Shirley, New York). Total work was determined as the work accomplished over 30 repetitions of maximal effort.

### Body Composition and Anthropometry Testing

Body composition and percent body fat were determined using dual-energy X-ray absorptiometry scans performed using the QDR 4500A whole body scanner (Hologic Inc., Bedford, Massachusetts). Weight was measured with on a calibrated electronic scale (GSE scale Systems, Novi Michigan). Waist circumference was measured by Gulick tape midway between the inferior border of the rib cage and the superior aspect of the iliac crest at the end of normal expiration.

### Blood Samples

Serum BDNF levels were measured by the Pennington Biomedical Research Center Clinical Research Laboratory at baseline and follow-up from archived blood samples stored at −80 degrees Celsius. Serum BDNF levels were analyzed using a Chemkine BDNF sandwich enzyme immunoassay (Milpore, Billerica, MA). The sensitivity of the assay was ±7.8 pg/mL with an inter-assay variation of ±8.5% and an intra-assay variation of ±3.5%. All samples for serum BDNF at baseline and follow-up were run on the same analysis plate in order to eliminate inter-assay variation between pre- and post-intervention pairs. HbA_1C_ was evaluated from venipuncture, and samples were run on Beckman Coulter Synchron LX automated analyzer (Fullerton, CA). At baseline and follow-up, blood samples were collected and processed after an overnight fast.

HART-D had separate intervention and assessment teams, and all assessment staff were blinded to participant randomization. The clinical testing and exercise training laboratories were in separate buildings, and participants were reminded frequently not to disclose their group assignment to assessment staff.

### Statistical Analysis

All data was analyzed using SAS version 9.1 (SAS Institute Inc, Cary, North Carolina). The final analysis of the present study was performed in adherent participants (70% adherent to exercise intervention consistent with the definition used in our main outcome paper [16]) with outliers removed. Outliers were defined as participants who were more than 95% above the median value for the change in serum BDNF levels following exercise training, and were removed from the final analysis. A one-way analysis of variance (ANOVA) was used to compare baseline characteristics between groups of continuous variables, and a chi-squared test (χ^2^) was used to compare baseline characteristics between categorical variables. Multiple linear regression was used to test the trend of baseline serum BDNF levels across quartiles of fitness, body composition, anthropometry, glucose control, and strength measures.

An analysis of covariance (ANCOVA) was used to determine the effect of exercise modality on serum BDNF levels with baseline value, age, sex, duration of diabetes, and ethnic group entered as covariates with outliers removed (N = 150). Results are presented as adjusted least square means with 95% confidence intervals (CIs). In addition, we performed the same analysis in all participants (outliers included) (N = 168). Spearman correlations were used to determine the association between the change in serum BDNF following exercise training and fitness, body composition, anthropometry, glucose control, and strength measures in exercisers only (N = 127). A *p* value of <0.05 was used as the criteria for statistical significance for all analyses.

## Results

Demographic data are presented in Table 1. The study sample had a mean (SD) age of 57.9 (8.3) yrs; a mean weight of 97.6 (19.5) kg; a mean BMI of 34.2 (6.1) kg/m^2^; was 57% female; and was ethnically diverse as approximately 35% of our study sample was Black. The average duration of diabetes in the sample was 7.3 yrs. (5.2), and approximately 99% of the population was currently taking diabetes related medication. No significant differences between randomization groups were present at baseline. Additionally, no significant differences in baseline serum BDNF levels between men (18034.5 pg/ml) and women (18048.0 pg/ml) (p = 0.991) or Blacks (18744.8 pg/ml) and Whites (17591.1 pg/ml) (p = 0.407) were observed.

**Table 1:** Baseline Characteristics.

	Control (N = 23)	Aerobic (N = 40)	Resistance (N = 44)	Combination (N = 43)
**Female % (n)**	69.6 (16)	57.5 (23)	52.3 (23)	55.8 (24)
**Ethnicity % (n)**				
**White**	52.2 (12)	62.5 (25)	63.6 (28)	65.1 (28)
**Black**	43.5 (10)	37.5 (15)	34.1 (15)	27.9 (12)
**Hispanic/Other**	4.4 (1)	0 (0)	2.3 (1)	7.0 (3)
**Age (yr)**	58.4 (8.7)	54.2 (7.3)	58.8 (8.7)	56.7 (8.2)
**Weight (kg)**	93 (19.4)	97.1 (19.9)	97.3 (17.2)	101.0 (21.4)
**BMI (kg/m2)**	33.3 (6.3)	33.9 (6.4)	33.8 (5.4)	35.4 (6.5)
**Waist circumference (cm)**	106.9 (13.3)	109.6 (14.7)	111.6 (12.6)	115.0 (15.4)
**Body fat (%)**	37.2 (6.9)	36 (7.7)	36.5 (7.9)	37.8 (7.1)
**Systolic blood pressure (mmHg)**	124.7 (13.9)	123 (11)	126 (13.0)	129.5 (12.3)
**Diastolic blood pressure (mmHg)**	75.5 (7.8)	76.2 (9.4)	74.2 (8.3)	74.7 (7.8)
**Hemoglobin A_1C_**	7.8 (1.7)	7.2 (1.1)	7.2 (1.0)	7.1 (1.1)
**Absolute VO_2peak_ (L/min)**	1.7 (0.5)	2.0 (0.5)	1.9 (0.5)	1.9 (0.5)
**Relative VO_2 peak_ (mL·kg^−1^·min^−1^)**	18.3 (4.1)	21.1 (5.3)	19.9 (4.5)	19.3 (3.7)
**Treadmill test duration (min)**	10.6 (2.3)	11.3 (3.2)	10.5 (2.7)	10.7 (2.4)
**Muscular work (Nm)**				
**Flexion**	1.2 (0.3)	1.1 (0.3)	1.1 (0.3)	1.1 (0.4)
**Extension**	1.2 (0.2)	1.1 (0.3)	1.3 (0.4)	1.2 (0.4)
**Diabetes duration (yrs)**	7.0 (5.4)	7.9 (6.0)	8.0 (5.6)	6.4 (3.6)
**Serum BDNF (pg/ml)**	16928.1 (6781.7)	18870.4 (8153.5)	17509.2 (8679.1)	18413.3 (7798.2)
**Diabetes medication % (n)**	100.0 (23)	97.5 (39)	100.0 (44)	100.0 (43)
**Biguanide %(n)**	60.9 (14)	70.0 (28)	59.1 (26)	72.09 (31)
**Sulfonylureas %(n)**	30.4 (7)	25.0 (10)	25.0 (11)	23.26 (10)
**Thiazolidnediones %(n)**	17.4 (4)	25.0 (10)	18.2 (8)	16.3 (7)
**Combo Class % (n)**	21.7 (5)	15.0 (6.0)	18.2 (8)	18.6 (8)
**Insulin % (n)**	26.1 (6)	15.0 (6)	15.9 (7)	20.9 (9)

### Serum BDNF levels at baseline across quintiles of cardiometabolic risk factors

Serum BDNF levels across quartiles of fitness, body composition, anthropometry, glucose control, and strength measures are shown in Table 2. Overall, no significant associations between these factors and baseline serum BDNF levels were observed (*all*, p>0.05). However, the trend for lower serum BDNF levels across quartiles of treadmill test duration approached significance (p-trend = 0.06). When analyses were performed separately in men and women, similar results were observed.

**Table 2:** The association between baseline serum BDNF levels (quartiles) and cardiometabolic risk factors.

	Q1 (<8603 pg/ml)	Q2 (8604–19922 pg/ml)	Q3 (19923–28747 pg/ml)	Q4 (>28747 pg/ml)	
	N = 38	N = 37	N = 38	N = 37	P-Trend
**Age (yrs)**	56.2 (6.9)	56.4 (9.3)	58.3 (8.3)	56.7 (8.8)	0.56
**Diabetes duration (yrs)**	8.3 (5.5)	6.4 (4.8)	7.7 (5.3)	6.9 (5.1)	0.44
**Relative VO_2 peak_ (mL·kg^−1^·min^−1^)**	20.1 (3.9)	19.9 (5.7)	19.0 (4.3)	20.3 (4.1)	0.53
**Absolute VO_2 peak_ (L/min)**	2.0 (0.5)	1.9 (0.6)	1.9 (0.4)	1.9 (0.6)	0.90
**Treadmill test duration (min)**	11.4 (2.5)	10.9 (3.1)	10.5 (2.6)	10.3 (2.4)	0.06
**Body mass index (BMI)**	34.7 (5.6)	34.2 (5.9)	35.0 (5.8)	32.9 (7.1)	0.35
**Waist circumference (cm)**	113.8 (14.6)	109.7 (13.6)	112.1 (12.9)	109.7 (16.0)	0.34
**Body fat (%)**	36.4 (6.2)	38.0 (8.1)	38.5 (7.9)	34.4 (6.8)	0.35
**Systolic blood pressure (mmHg)**	127.8 (12.2)	124.5 (13.1)	125.6 (11.5)	126.2 (13.8)	0.68
**Diastolic blood pressure (mmHg)**	74.8 (10.4)	75.2 (8.0)	74.6 (7.6)	75.7 (7.4)	0.74
**HbA_1C_ (%)**	7.4 (1.1)	7.0 (1.3)	7.6 (1.4)	7.1 (1.1)	0.90
**Work flexion (Nm)**	1032.7 (436.8)	975.2 (492.0)	970.0 (553.1)	1038.3 (562.6)	0.98
**Work extension (Nm)**	2506.0 (790.1)	2392.8 (990.5)	2250.5 (946.4)	2411.6 (957.9)	0.52

Multiple linear regression was used to test the trend of baseline BDNF. P-trend <0.05 was the criteria for statistical significance.

### The effects of exercise training modality on serum BDNF levels

**Figure 2:**
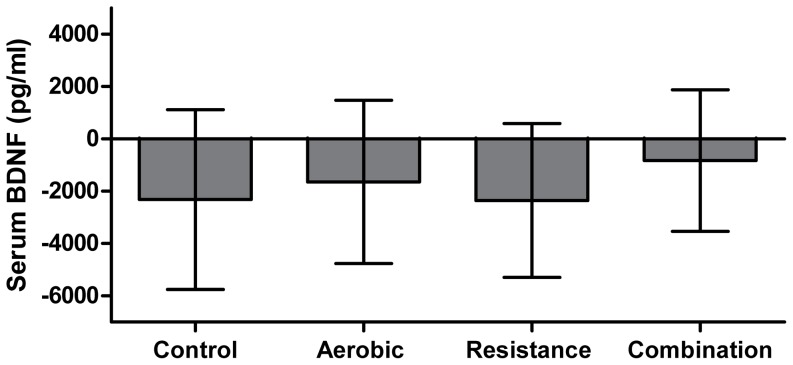
The effect of exercise training modality on serum BDNF levels. Results are presented as adjusted least square means with 95% confidence intervals. The statistical model is adjusted for baseline value, age, sex, duration of diabetes, and ethnic group.

The effects of exercise modality on serum BDNF levels are shown in Figure 2. No significant change in serum BDNF levels was observed following exercise training in the aerobic (−1649.4 pg/ml, CI: −4768.9 to 1470.2), resistance (−2351.2 pg/ml, CI:−5290.7 to 588.3), or combination groups (−827.4 pg/ml, CI: −3533.3 to1878.5) compared to the control group (−2320.0 pg/ml, CI: −5750.8 to 1110.8). Similarly, when the same analysis was performed with outliers included (N = 168), no significant change was observed in serum BDNF levels between the aerobic (−432.9 pg/ml, CI: −432.9 pg/ml, CI: −4413.9 to 3548.2), resistance (−827.9 pg/ml, CI: −4601.9 to 2946.2), and combination groups (−1912.5 pg/ml, CI: −5257.3 to 1432.2) compared to the control group (−3864.1 pg/ml, CI: −8487.3 to −759.2).

### Correlations of changes in serum BDNF levels with changes in cardiometabolic risk factors following exercise training

Spearman correlations for change in serum BDNF levels following exercise training (exercisers only) with cardiometabolic risk factors are shown in Table 3. Change in serum BDNF levels following exercise training was directly associated with waist circumference (r = 0.25, p = 0.005) but not associated with changes in systolic blood pressure, diastolic blood pressure, weight, fitness, or strength measures (p>0.05).

**Table 3:** Spearman correlations for the change in BDNF following exercise training with changes in cardiometabolic risk factors in exercisers (N = 127).

	*r*	p-value
Δ Relative VO_2 peak_ (mL·kg^−1^·min^−1^)	0.02	0.84
Δ Absolute VO_2 peak_ (L/min)	0.03	0.75
Δ Treadmill test duration (min)	−0.11	0.21
Δ Body mass index (BMI)	0.10	0.28
Δ Waist circumference (cm)	0.25	0.005
Δ Body fat (%)	0.10	0.26
Δ Systolic blood pressure (mmHg)	−0.07	0.39
Δ Diastolic blood pressure (mmHg)	−0.02	0.81
Δ HbA_1C_ (%)	0.02	0.86
Δ Work flexion (Nm)	−0.02	0.85
Δ Work extension (Nm)	−0.01	0.94

## Discussion

The primary finding from the present study was cardiometabolic risk factors were not associated with serum BDNF levels at baseline in individuals with type 2 diabetes. Furthermore, 9 months of aerobic, resistance, or combination training did not alter serum BDNF levels compared to control participants, but reductions in waist circumference were associated with decreased serum BDNF following exercise training. To our knowledge, this is the first large randomized controlled study comparing the effects of aerobic, resistance, and combination training on serum BDNF levels in individuals with type 2 diabetes.

Currently, data are limited that examine the implications of cardiometabolic risk factors on BDNF in individuals with type 2 diabetes. Suwa et al. [9] found that in a small sample (n = 24) of newly diagnosed females with type 2 diabetes that serum BDNF levels were elevated compared to healthy women (n = 7). The authors further reported a direct relationship between BMI (r = 0.54, p = 0.006), body fat (r = 0.55, p = 0.004), triglycerides (r = 0.47, p = 0.019), fasting blood glucose (r = 0.44, p = 0.032), and HOMA-IR (r = 0.51, p = 0.011) with serum BDNF values. Similarly, Krabbe et al. [7] found that plasma BDNF levels were the lowest in obese individuals with type 2 diabetes compared to individuals with impaired glucose tolerance and normal glucose tolerance.

In the present study, no associations were found at baseline between fitness, anthropometry, body composition, strength, or glucose control measures and serum BDNF levels in individuals with type 2 diabetes. However, the relationship between serum BDNF levels and longer treadmill test duration approached significance. Our results are supported by data from Fujinami et al. [6] who found that serum BDNF was not associated with age, BMI, blood pressure, diabetes duration, or HbA_1C_ in Japanese men and women with type 2 diabetes. Differences between our study and previous studies finding an association between BDNF levels and cardiometabolic risk factors in individuals with type-2 diabetes may be due to the differences in the type of sample analyzed for BDNF (plasma vs. serum), differences in study population (HART-D: 34% Black), and possible differences in the use of diabetes medications.

Several studies have demonstrated an increase in serum BDNF levels following acute exercise [27]–[31]; however, the effects of chronic exercise on BDNF remain unclear. Zoladz et al. [19] found an increase in plasma BDNF levels in young men following 5 months of moderate intensity aerobic exercise training. Castellano et al. [20] found that serum BDNF levels significantly increased in individuals with multiple sclerosis following 4 weeks of aerobic exercise training but returned near baseline levels following 8 weeks of training. Erickson et al. [23] conducted a large randomized controlled trial in older adults without dementia and found no significant change in BDNF following 6 months of aerobic exercise. The only study investigating the effects of aerobic versus resistance training was conducted by Schiffer et al. [21], which found no significant change in plasma BDNF following aerobic or resistance exercise in healthy young adults.

The present study is the first to our knowledge to evaluate the effects of exercise training on serum BDNF specifically in individuals with type 2 diabetes or to directly compare the effect of different exercise modalities in a large randomized trial. We found no significant change in serum BDNF following aerobic, resistance or combination exercise training compared to control participants. Additionally, we found that reductions in waist circumference following exercise training in exercisers were positively associated with serum BDNF levels, suggesting that reductions in central adiposity may be involved in the regulation of BDNF. The physiologic importance of these findings on cognitive function should be addressed in future investigations since cognitive measures were not collected in HART-D. Changes in fitness, body composition, glucose control, and strength measures were not associated with changes in serum BDNF following exercise training in the present study.

The strengths of this investigation include a randomized study design with a large diverse study population. Additionally, all exercise training sessions were supervised by study staff, controlled for caloric expenditure, and all exercise groups exercised for a similar amount of time. Limitations of the present study are that it is a post-hoc analysis from archived blood samples, and we did not collect any measures of cognitive function. Last, since blood samples were collected at only baseline and follow-up, we cannot evaluate the time course of the changes in serum BDNF during the exercise intervention.

In conclusion, the results of the present study found that serum BDNF levels at baseline were not associated with fitness, body composition, anthropometry, glucose control, and strength measures in individuals with type 2 diabetes. In addition, no significant change in serum BDNF levels was observed compared to control participants following 9 months of exercise irrespective of training modality. Future research should evaluate the relationship between serum BDNF levels with memory and cognitive function specifically in individuals with type 2 diabetes, and the physiological mechanisms responsible for the association between exercise training related changes in central adiposity and serum BDNF levels.
